# Development of Edible Coating from Gelatin Composites with the Addition of Black Tea Extract (*Camellia sinensis*) on Minimally Processed Watermelon (*Citrullus lanatus*)

**DOI:** 10.3390/polym14132628

**Published:** 2022-06-28

**Authors:** Salwa Salsabiela, Ambar Sukma Sekarina, Hanifa Bagus, Aulia Audiensi, Farah Azizah, Windy Heristika, Manikharda, Eko Susanto, Heli Siti Halimatul Munawaroh, Pau Loke Show, Andriati Ningrum

**Affiliations:** 1 Department of Food and Agricultural Product Technology, Faculty of Agricultural Technology, Universitas Gadjah Mada, Flora Street No. 1, Bulaksumur, Yogyakarta 55281, Indonesia; salwasalsabiela@ugm.ac.id (S.S.); ambarnana@gmail.com (A.S.S.); hanifabagus@gmail.com (H.B.); auliaaudiensi@gmail.com (A.A.); farahazizah@gmail.com (F.A.); windyheristika@gmail.com (W.H.); manikharda@gmail.com (M.); 2 Department of Fish Products Technology, Faculty of Fisheries and Marine Science, Universitas Diponegoro, Jl. Prof. Soedarto SH Kampus Tembalang, Semarang 50275, Indonesia; ekothp@live.undip.ac.id; 3 Study Program of Chemistry, Department of Chemistry Education, Faculty of Mathematics and Science Education, Universitas Pendidikan Indonesia, Bandung 40154, Indonesia; heli@upi.edu; 4 Department of Chemical and Environmental Engineering, University of Nottingham Malaysia, Jalan Broga 43500, Selangor, Malaysia; pauloke.show@nottingham.edu.my

**Keywords:** tuna skin by-product, fresh-cut watermelon, extract tea, edible coating

## Abstract

The purpose of this research was to determine the effect of composite fish gelatin–chitosan edible coatings enriched with black tea extract on the physical, chemical, and fungal decay properties of minimally processed watermelons stored at ±4 °C for 13 days. In this study, tuna skin gelatin was extracted and used to prepare edible coating solutions which comprised 4% gelatin, 2% chitosan, 2% calcium lactate, 2% glycerol, and black tea extract (0%; 0.25%; 0.50%; 0.75%; 1%). The samples were coated using the layer-by-layer dipping technique. This study showed that composite fish gelatin–chitosan edible coating enriched with black tea extract maintained and improved weight loss, texture (hardness), color, pH, and total soluble solid antioxidant activity and prevented fungal decay on minimally processed watermelons stored at ±4 °C for 13 days. The development in this study of edible film and a coating prepared from fish gelatin–chitosan and the incorporation of black tea extract as an antioxidant or antimicrobial agent can be a new approach to preventing postharvest loss and increasing the shelf life of minimally processed watermelon.

## 1. Introduction

It is hoped that the consumption of sufficient fruits and vegetables during the current pandemic can have many functional effects on human health [1,2]. In Indonesia, fruit consumption is less than vegetable consumption. One of the reasons for the low consumption of fruit is the necessity to peel it before consumption. Therefore, there is the potential to develop minimally processed fruit because it only involves washing, peeling, cutting, packaging, and storing the fruit at low temperatures to maintain the freshness and nutritional content [2]. An example of minimally processed fruit that is often found in the market is watermelon (*Citrullus lanatus*).

Watermelon is very popular with the public because of its sweet taste and high water content, which give it a freshness when consumed. Watermelon is rich in vitamins A, B6, C, and K and antioxidants, which are very good for maintaining a healthy body [1,3]. Currently, many watermelons are sold in a minimally processed form. Minimally processed watermelon, in the form of slices without the skin, is more in demand by the public because of the convenience and practicality of consuming it. However, minimally processed watermelon easily loses weight through the evaporation of its water content, the growth of spoilage microbes, and the several enzymatic reactions that cause changes in the texture, color, taste, and nutritional content [3]. One way to maintain quality and freshness and extend the shelf life of minimally processed watermelon is to coat the pieces of fruit using a coating solution that is safe for consumption, commonly called edible coating [4,5].The edible coating can maintain quality and extend the shelf life of minimally processed fruit. The requirements for the components that can be used as edible coatings include those that do not affect the smell and taste of the food used and are easy to obtain, easy to digest, and non-toxic [5,6]. The edible coating could also contain several polymers from volatile or non-volatile parts [7]. Edible coating is used for the coating of fresh-cut fruits and vegetables such as watermelon. The sample is immersed in the film-forming solution, and it creates a protective coating directly on the surface of food such as minimally processed fruits [8]. The polymers that are usually used for edible coating could be from polymers of protein, carbohydrates, and lipid derivatives [8,9]. Gelatin is one of the ingredients that is often used as a component of edible coatings. One type of fish that is effective in producing gelatin is tuna. Gelatin from tuna fish skin as a food by-product has a good gel strength and viscosity [8,9]. Several studies have succeeded in combining gelatin with chitosan as a composite edible coating. Chitosan is one of the polysaccharides that is widely used as a constituent of edible coatings because it has antimicrobial properties. The combination of gelatin and chitosan can increase the antimicrobial properties of chitosan [10,11].

The addition of natural extracts can also improve the physical and functional properties of edible coatings, especially natural extracts that are rich in bioactive compounds [12]. Tea (*Camellia sinensis*) is a plant that has both antimicrobial and antioxidant activity [13]. Black tea contains tannins such as thearubigins, which have antimicrobial activities. On the other hand, several polyphenolic compounds, such as catechins, theaflavins, and methyl jasmonate, also have antimicrobial and antioxidant activity [14,15]. The fermentation process in black tea converts polyphenolic compounds (catechins and their derivatives) into theaflavins and thearubigins so that the content of catechin compounds decreases. However, many studies have shown that the antioxidant activity of black tea is comparable to that of green tea [12,14,15]. The theaflavins in black tea have the same antioxidant potential as the catechins in green tea [14,16]. The theaflavins in black tea extract are more effective in capturing free radicals than the catechins found in green tea. Therefore, based on the description above, in our study the aim was to determine the effect of using tuna skin gelatin composite and chitosan enriched with the addition of black tea extract as an edible coating on minimally processed fruits, e.g., fresh-cut watermelon. The samples were stored at ±4 °C for 13 days at low-temperature storage (±4 °C), and the physicochemical characterization and the fungal decay of the minimally processed watermelon was evaluated.

## 2. Material and Methods

### 2.1. Materials

The ingredients used in this study include red seeded watermelon 7–8 days after harvest (Berkat fruit shop, Yogyakarta, Indonesia), black tea (Tong Tji Super), yellowfin tuna (*Thunnus albacares*) skin (Omah Tuna, Yogyakarta, Indonesia), chitosan with a 20–100 mPas viscosity and a min 94% degree of acetylation (Phy Edumedia, Malang, Indonesia), glycerol (Progo Mulyo, Yogyakarta, Indonesia), aquades (Progo Mulyo, Yogyakarta, Indonesia), sodium hydroxide (Merck, Darmstadt, Germany), sulfuric acid (Merck, Darmstadt, Germany), citric acid (Merck, Darmstadt, Germany), acetic acid (Merck, Darmstadt, Germany), methanol (Merck, Darmstadt, Germany), and DPPH (2,2-diphenyl-1-picrylhydrazyl) (Sigma Aldrich, Singapore).

### 2.2. Extraction of Gelatin from Yellowfin Tuna Fish Skin

The extraction of the gelatin began with the separation of the tuna skin from the scales, bones, and nonskin components, followed by the cutting of the tuna skin to a size of ±5 × 5 cm. The tuna skin was soaked in NaOH solution with a 0.2% ratio of 1:6 (*w*/*v*) for 2 h and neutralized to pH 6–7 with water. The fish skin was then immersed in a 0.2% H_2_SO_4_ solution at a ratio of 1:6 (*w*/*v*) for 2 h, washed to neutralize, soaked in citric acid (C_6_H_8_O_7_) 0.1%, with a ratio of 1:6 (*w*/*v*) for 2 h, and washed to neutral pH (6–7). Continued extraction was performed with distilled water in a 60 °C water bath shaker for 6 h at a ratio of 1:3 (*w*/*v*) and filtered twice before drying at a temperature of 50 °C for 24 h [17]. The gelatin sheet was then milled to obtain the gelatin powder [17]. The specification of gelatin viscosity is approximately 25 cPs.

### 2.3. Preparation of Edible Coating Solution

The process of applying the edible coating solution onto the watermelon (*Citrullus lanatus*) was achieved using a layer-by-layer technique, with three different types of edible coating solutions. The first solution was a gelatin–chitosan composite solution and glycerol; the second solution was a black tea extract at five concentration variations (0%; 0.25%; 0.50%; 0.75%; and 1%); and the third solution was a 2% calcium lactate solution.

### 2.4. Coating of Edible Coating Solution on Minimally Processed Watermelon (Citrullus lanatus)

**Figure 1:**
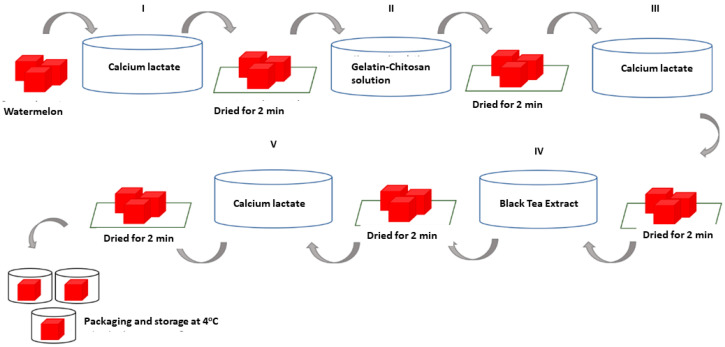
Layer-by-layer technique of edible coating.

The process of applying the edible coating solution to the minimally processed watermelon (*Citrullus lanatus*) was carried out using the layer-by-layer immersion technique. First, a sample of watermelon that had been minimally processed with a size of ±(3 × 3 cm) was immersed in a 2% calcium lactate solution for two minutes, then drained and dried for two minutes. Next, the sample was immersed in a gelatin–chitosan composite solution and glycerol for two minutes, then drained and dried for two minutes. After that, the sample was again immersed in a 2% calcium lactate solution for two minutes, then drained and dried for two minutes. Furthermore, the packaging was carried out in plastic cups for samples treated with 0% black tea extract, while the other samples were immersed in a black tea extract solution with different concentrations, namely 0.25%, 0.50%, 0.75%, and 1% each for two minutes, then drained and dried for two minutes. After that, the sample was again immersed in a 2% calcium lactate solution for two minutes, then drained and dried for two minutes. Then, the sample was put into a plastic cup and stored in the refrigerator at a temperature of ± 4 °C. The sample was stored for 13 days of storage, and the physicochemical characterization and fungal decay evaluation were investigated at 4, 7, 10, and 13 days of storage. The following is a schematic of the edible coating solution coating process on watermelons that are minimally processed with the layer-by-layer technique. Further processes of the layer-by-layer technique can be seen in Figure 1.

### 2.5. Weight Loss Analysis

Weight loss analysis was carried out gravimetrically, namely by calculating the difference in weight before and after storage [18,19]. The measurement of the sample weight was carried out using an analytical balance every three days for 13 days of storage.

(1) $$\mathrm{Weight}\;\mathrm{loss}\;\left(\%\right)=\frac{\left(\mathrm{initial}\;\mathrm{weight}\;\left(\mathrm{grams}\right)-\mathrm{final}\;\mathrm{weight}\;\left(\mathrm{grams}\right)\right)}{\left(\mathrm{initial}\;\mathrm{weight}\;\left(\mathrm{grams}\right)\right)}\times 100\%$$

### 2.6. Texture Analysis

The texture parameter observed was hardness. Texture analysis was performed using a Universal Testing Machine (UTM) with a pre-load of 0.02 N; a pre-load speed of 50 mm/min; and a test speed of 50 mm/min. The analysis of sample hardness was interpreted by the maximum force (Fmax) required to pierce 30% of the sample height. Higher Fmax (Newton) values indicate that the texture of the sample is becoming harder [17].

### 2.7. Color Analysis

Color intensity analysis was carried out using a Minolta CR-400 Chroma Meter (Konica Minolta, Inc., Tokyo, Japan). The sample was placed on top of the chroma meter sensor, then a light was fired at the part to be measured so that the values of L (lightness), a (green-red chromaticity), and b (yellow-blue chromaticity) would appear on the chroma meter display [17].

### 2.8. pH Analysis

The pH analysis was performed using a pH meter previously calibrated with standard buffer solutions of pH 4 and 7. A total of 10 g of the sample was mashed using a blender and then centrifuged for one hour at a speed of 1000× *g* at 4 °C until separation between the natant and the supernatant occurred. The supernatant was used for pH analysis. To measure the pH value, the pH meter probe was immersed in the sample supernatant to obtain the pH value directly [20].

### 2.9. Total Dissolved Solids Analysis

Total dissolved solids analysis was performed using an Atago Master-53M refractometer (Atago Co., Ltd., Fukuoka, Japan). Sample preparation for the total dissolved solids analysis was carried out as in step 2.8. One to two drops of the sample supernatant at room temperature were placed on the prism of the refractometer, then the Brix percentage was read through the eyepiece of the refractometer [21].

### 2.10. Antioxidant Activity Analysis

A total of 15 g of watermelon samples were mashed using a blender for 2 min, then centrifuged for one hour at a speed of 1000× *g* at 4 °C until the separation between the natant and the supernatant occurred. Then, the natant or solid particles resulting from the centrifugation were filtered using a vacuum filter.

For the analysis of the antioxidant activity, a sample solution with a concentration of 100,000 ppm was made by dissolving 2.5 g of the sample, which had been filtered with a vacuum filter in 25 mL of methanol, then stirred with a magnetic stirrer for 90 min. After that, the samples were filtered with filter paper; then, 1 mL of each was taken and put into a test tube. Each sample had 7 mL of 0.1 mM DPPH solution added and homogenized using a vortex. The samples were then incubated for 30 min in the dark. After that, the absorbance value was measured using a UV-Vis spectrophotometer at a wavelength of 517 nm.

The antioxidant activity of the sample is interpreted in terms of the percentage of radical scavenging activity, namely the ability of the sample to capture free radical compounds. The more free radical compounds that can be captured, the more the antioxidant activity content in the sample [17].

(2) $$\mathrm{DPPH}\;\mathrm{radical}\;\mathrm{scavenging}\;\mathrm{activity}\;\left(\%\right)=\frac{\left(A\;\mathrm{blank}-A\;\mathrm{sample}\right)\;}{\left(A\;\mathrm{blank}\right)}\times 100\%$$

where A = Absorbance.

### 2.11. Fungal Decay

Determination of fungal decay was performed by observing the presence or absence of the fungi that grow on the surface of the sample, then calculating the percentage of the sample that was overgrown with fungus (Equation (3)). The sample is considered damaged if there is fungal mycelium on its surface. The results of these observations are expressed as the percentage of samples contaminated with fungi [22].

(3) $$\mathrm{Fungal}\;\mathrm{contamination}\;\left(\%\right)=\frac{\left(\mathrm{Number}\;\mathrm{of}\;\mathrm{samples}\;\mathrm{contaminated}\;\mathrm{with}\;\mathrm{fungus}\right)}{\left(\mathrm{Total}\;\mathrm{number}\;\mathrm{of}\;\mathrm{samples}\right)}\times 100\%$$

### 2.12. Statistical Analysis

The statistical analysis was performed using Minitab v. 19 statistical software (State College, PA, USA). Analysis of variance (ANOVA) and Tukey’s pairwise comparisons were carried out using a level of 95% confidence. The experiments were performed at least in triplicate, and the data were reported as mean ± standard deviation.

## 3. Results and Discussion

### 3.1. Weight Loss

**Figure 2:**
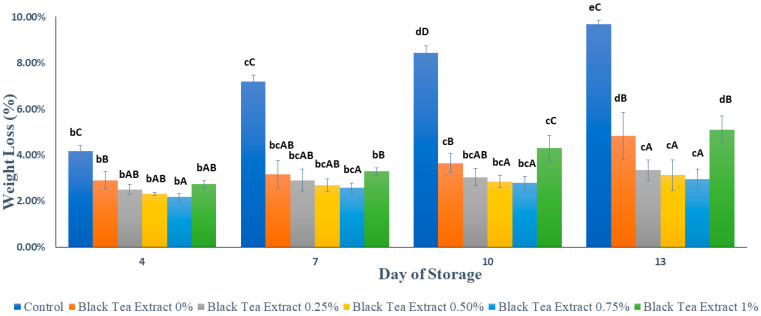
Effect of an edible coating of gelatin composite enriched with black tea extract on weight loss (%) of minimally processed watermelon during storage. The value shown is the average of the three experimental replications. ^A–D^ Values followed by the same capital letters showed no significant difference (*p* > 0.05) between treatments on the same day. ^b–e^ Values followed by the same non-capital letters showed no significant difference (*p* > 0.05) between storage times in the same treatment.

Figure 2 shows that the application of an edible coating of gelatin composite enriched with black tea extract can effectively reduce the percentage of weight loss in minimally processed watermelon stored at ±4 °C for 13 days. The samples not treated with an edible coating (control) had a significantly higher percentage of weight loss (*p* < 0.05) than the samples treated with an edible coating (Figure 1). Weight loss is generally solely attributed to water loss, although the loss of some other components may also contribute to this problem. Nevertheless, except for water loss, the contribution from other components is considered negligible. This loss of water decreases the turgor and firmness of fruits. It can cause acceleration of surface depression and deformation of the produce. Water loss is associated with several other changes occurring in fruits and can act as a trigger to initiate these changes [23]. The edible coatings cover the surface layer of the fruit and inhibit the processes of respiration, transpiration, and syneresis [21,24,25]. This finding is evidence for the benefits of applying the edible coating to fresh-cut watermelon pieces, mainly due to the formation of a polymeric barrier that can reduce the water loss from fresh-cut samples, as is found with other fruits [3,22].

Based on Figure 2, the higher the concentration of black tea extract added, the lower the percentage of weight loss. The addition of black tea extract as a natural bioactive compound can act as a cross-linking agent in edible coating components to increase the barrier against water vapor because gelatin is sensitive to water vapor when used as the sole constituent of edible coatings [24]. Although there was a decrease in the percentage of weight loss, there was no significant difference (*p* > 0.05) in the addition of 0.25–0.75% black tea extract. However, there was an increase in weight loss with the addition of 1% black tea extract. This could be caused by the addition of phenolic compounds to a certain level that can reduce the gel properties of gelatin. Phenolic compounds are more likely to interact with gelatin as an aggregate, causing an irregular gel structure [20,24].

Based on Figure 2, the concentration of black tea extract, storage time, and the interaction between the two treatments had a significant effect (*p* < 0.05) on the percentage of minimally processed watermelon weight loss during storage. Our results showed that there was no significant difference in the percentage of weight loss (*p* > 0.05) in the treatment with the addition of 0.25% black tea extract or with 0.50% and 0.75% on the 13th day. Therefore the treatment of adding black tea extract with a concentration of 0.25% to the edible coating solution of the gelatin composite was the best in reducing the percentage of weight loss of watermelons processed for at least 13 days of storage at a temperature of ±4 °C.

### 3.2. Texture

**Figure 3:**
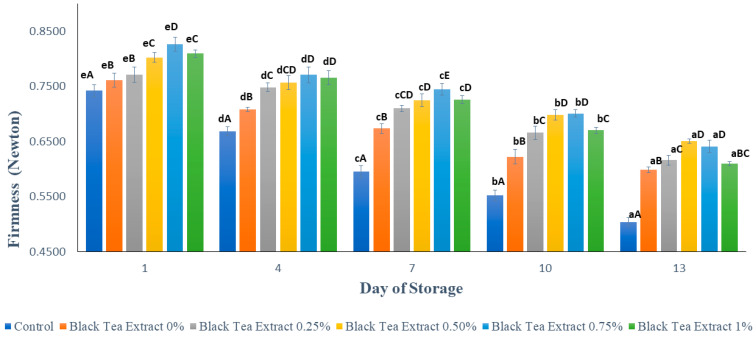
Effect of edible coating of gelatin composite enriched with black tea extract on the hardness (Newton) of minimally processed watermelon during storage. The value shown is the average of the three experimental replications. ^A–E^ Values followed by the same capital letters showed no significant difference (*p* > 0.05) between treatments on the same day. ^a–e^ Values followed by the same non-capital letters showed no significant difference (*p* > 0.05) between storage times in the same treatment.

Figure 3 shows the results of texture analysis (firmness) on minimally processed watermelon coated with an edible coating of a gelatin composite enriched with black tea extract. The higher the Fmax (Newton) value, the harder the sample texture. Based on Figure 3, the longer the storage time, the lower the Fmax value, but the edible coating treatment can reduce the loss of firmness. Firmness is one of the most important quality attributes for all fruits, although it may be of more critical importance to some fruits (such as berries) than others. Tissue softening is thus a severe concern that affects visual quality and reduces the shelf life of fruits, especially in fresh-cut products [23]. The control sample had a significantly lower firmness (*p* < 0.05) than the sample treated with an edible coating. The hardness of the watermelon samples was minimally reduced during storage, but the samples treated with an edible coating of sodium alginate had a significantly higher hardness (*p* < 0.05) than the control. This is because during storage there are still metabolic processes in fruit, such as respiration and transpiration, as well as the activity of pectinase enzymes such as polygalacturonase and pectin methylesterase, which can convert insoluble pectin into water-soluble pectin so that the fruit texture becomes soft [3,22].

Based on Figure 3, the higher the concentration of black tea extract added, the greater the Fmax value, but there was a decrease with the addition of 1% black tea extract. The addition of black tea extract can act as a cross-linking agent that can increase hardness at certain concentrations. The hardness of the fruit can be related to the percentage of weight loss because the higher the percentage of weight loss (loss of water), the softer the texture of the fruit. The incorporation of tea extract into coating formulations for fruits and vegetables may have a remarkable effect on the firmness of the tissue. The presence of some compounds in tea extract may have some activity that promotes alterations in firmness. In addition, the use of calcium lactate also plays a role in maintaining fruit texture, because calcium ions can strengthen cell walls. It can be seen in Figure 3 that on days 10 and 13 there was no significant difference in hardness (*p* > 0.05) with the addition of 0.50% and 0.75% black tea extract.

### 3.3. Color

#### 3.3.1. Lightness

Visual appearance and color are important quality criteria that directly influence the customer’s perception of quality and are two of the most crucial quality attributes in fruits. The color of fruits particularly changes in their storage; when transformed into fresh-cut products they become highly prone to developing a browning discoloration. This discoloration in fruits is thus a critical issue that may render the product unacceptable to the consumers [23].

**Figure 4:**
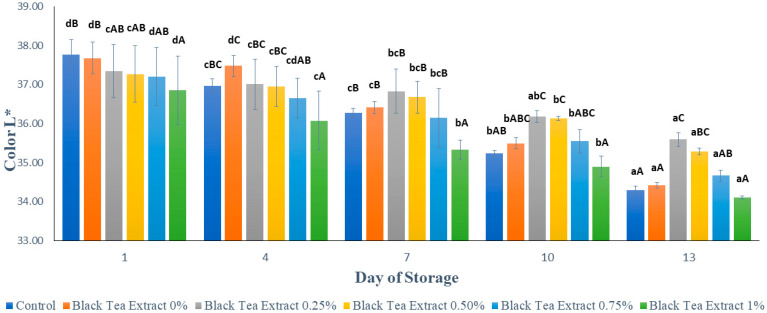
Effect of edible coating of gelatin composite enriched with black tea extract on the color (L*) of minimally processed watermelon during storage. The value shown is the average of the three experimental replications. ^A–C^ Values followed by the same capital letters showed no significant difference (*p* > 0.05) between treatments on the same day. ^a–d^ Values followed by the same non-capital letters showed no significant difference (*p* > 0.05) between storage times in the same treatment.

Figure 4 shows the results of the brightness analysis (L*) of minimally processed watermelon coated with an edible coating of a gelatin composite enriched with black tea extract. Based on Figure 3, the surface brightness value of the processed watermelon samples minimally decreased during storage. This can be caused by the activity of the PPO (polyphenol oxidase) enzyme which causes a decrease in brightness on the surface of the fruit flesh and tends to brown [25]. Browning reactions can occur because oxygen reacts directly with the polyphenol compounds catalyzed by the polyphenol oxidase enzyme to form a brown melanin compound. Oxygen can react directly with polyphenol compounds if some cells or tissues are open due to wounds [26]. Enzymatic browning diminishes visual appearance, makes undesirable changes in flavor, and causes a loss of nutrients in fresh/fresh-cut fruits and vegetables. Such changes make the product less desirable to the consumers. The development of browning degrades the original color of the product [2]. In the control, however, the decrease in brightness value tends to be greater than the sample treated with the edible coating because the edible coating can reduce the contact between oxygen and the polyphenol compounds. Gelatin and chitosan, as components of edible coatings, have good barrier properties against oxygen [4,27].

Based on Figure 4, on the first day, the higher the concentration of black tea extract added, the darker the color of the black tea extract solution used as an edible coating and therefore the lower the brightness value of the sample. However, during storage, the edible coating treatment enriched with black tea extract was better able to maintain brightness than the control. It can be seen in Figure 4 that on day 13 there was no significant difference in brightness value (*p* > 0.05) in the control treatment or in the edible coatings with the addition of 0% and 1% black tea extract. The application of an edible coating incorporated with green tea has been applied to fresh lettuce. The treatments prevented ascorbic acid and carotenoid loss. Green tea has also been incorporated into the coating solution applied to peach slices to enhance their shelf life [2].

The results of the statistical analysis showed that the concentration of black tea extract, storage time, and the interaction between the two treatments had a significant effect (*p* < 0.05) on the brightness of the watermelon flesh that was minimally processed during storage. Further Duncan’s test results showed that there was no significant difference in brightness (*p* > 0.05) with the addition of 0.25% and 0.50% black tea extract treatment on day 13. Therefore, the addition of black tea extract with a concentration of 0.25% in the edible coating solution of gelatin composite is the best treatment for maintaining the brightness of the color of watermelons processed for at least 13 days of storage at a temperature of ± 4 °C.

#### 3.3.2. a (Degree of Redness)

**Figure 5:**
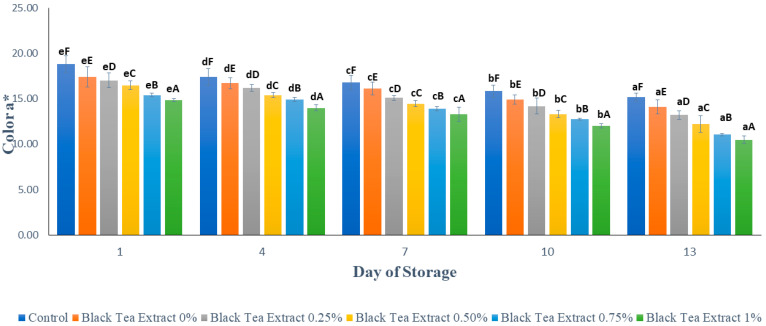
Effect of edible coating of gelatin composite enriched with black tea extract on the color (a*) of cut watermelon during storage. The value shown is the average of the three experimental replications. ^A–F^ Values followed by the same capital letters showed no significant difference (*p* > 0.05) between treatments on the same day. ^a–e^ Values followed by the same non-capital letters showed no significant difference (*p* > 0.05) between storage times in the same treatment.

Figure 5 shows the results of the analysis of the reddish color (a*) of minimally processed watermelon coated with an edible coating of a gelatin composite enriched with black tea extract. Based on Figure 5, the red color of all the samples of minimally processed watermelon experienced a significant decrease (*p* < 0.05) during the storage period. Lycopene is a red pigment in watermelon that has the ability to be an antioxidant; so, it is sensitive and easily damaged when exposed to light and oxygen during storage [1]. Samples without treatment (control) had a higher intensity of red color than samples treated with an edible coating. This can be caused by the red pigment in watermelon dissolving during the drying process in an edible coating solution. There was a significant difference (*p* < 0.05) for each addition of black tea extract concentration to the red color of minimally processed watermelon samples. Black tea contains theaflavin and thearubigin compounds, which give the tea a brown color so that the higher the concentration of black tea extract added, the more theaflavin and thearubigin compounds are present, which will affect the appearance of color in minimally processed watermelon samples [14].

#### 3.3.3. Degree of Yellowness

**Figure 6:**
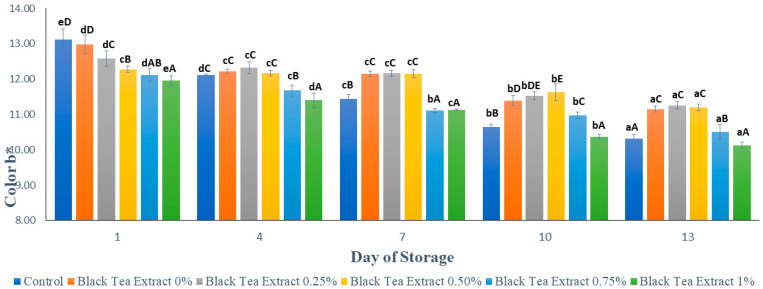
Effect of edible coating of gelatin composite enriched with black tea extract on the color (b*) of minimally processed watermelon during storage. The value shown is the average of the three experimental replications. ^A–E^ Values followed by the same capital letters showed no significant difference (*p* > 0.05) between treatments on the same day. ^a–e^ Values followed by the same non-capital letters showed no significant difference (*p* > 0.05) between storage times in the same treatment.

Figure 6 shows the results of the analysis of the yellowish color (b*) on minimally processed watermelon coated with an edible coating of a gelatin composite enriched with black tea extract. Based on Figure 6, the yellow color in all the samples of minimally processed watermelon tends to decrease during the storage period. This can be seen in Figure 6, where, on the first day, the higher the concentration of the addition of black tea extract, the lower the intensity of the yellow color. On the first day, the sample without treatment (control) had a higher yellow color intensity than the sample treated with an edible coating, but there was a significant decrease (*p* < 0.05) during storage so that the control had the lowest yellow color intensity on the 13th day. However, on day 13 there was no significant difference (*p* > 0.05) between the control and the treatment with the addition of 1% black tea extract.

### 3.4. pH

**Figure 7:**
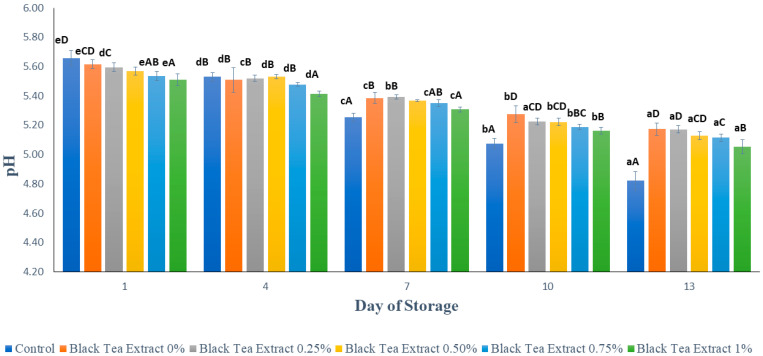
The effect of edible coating from gelatin composite enriched with black tea extract on the pH of watermelon was minimally processed during the storage period. The value shown is the average of the three experimental replications. ^A–D^ Values followed by the same capital letters showed no significant difference (*p* > 0.05) between treatments on the same day. ^a–e^ Values followed by the same non-capital letters showed no significant difference (*p* > 0.05) between storage times in the same treatment.

Figure 7 shows the results of the pH analysis on minimally processed watermelon coated with an edible coating of a gelatin composite enriched with black tea extract. Based on Figure 7, on the first day the sample without treatment (control) had the highest pH value compared to the sample with the edible coating treatment, and the higher the black tea extract added to the edible coating solution, the lower the pH of the sample. This could be caused by the use of 1% acetic acid to dissolve chitosan as a component of the edible coating composites and by the presence of organic acids in tea, such as malic acid, citric acid, oxalic acid, and succinic acid, which affect the pH of the sample [15].

There was a decrease in pH in all the treatments during the storage period. This was presumably due to the activity of the microorganisms that can convert sugar into organic acids. It can be seen in Figure 6 that the decrease in pH in samples treated with an edible coating enriched with the addition of black tea extract was not as sharp as in the control, because chitosan and black tea, as components of edible coatings, have antimicrobial activity, and the combination of gelatin and chitosan can increase the antimicrobial properties of chitosan [22]. Fruits are a naturally existing source of several organic acids such as Vitamin C. Vitamin C (ascorbic acid) degradation occurs during storage because it is highly sensitive and is lost upon exposure to heat, light, oxygen metals, and enzymes. Coating treatments have been shown to reduce the loss of organic acids such as ascorbic acid in fresh/fresh-cut fruits. Usually, ascorbic acid content decreases with the storage of fruits, irrespective of whether the fruit is covered with a coating or not, but the extent of the loss may be less in fruits that are coated [23].

### 3.5. Total Soluble Solid

**Figure 8:**
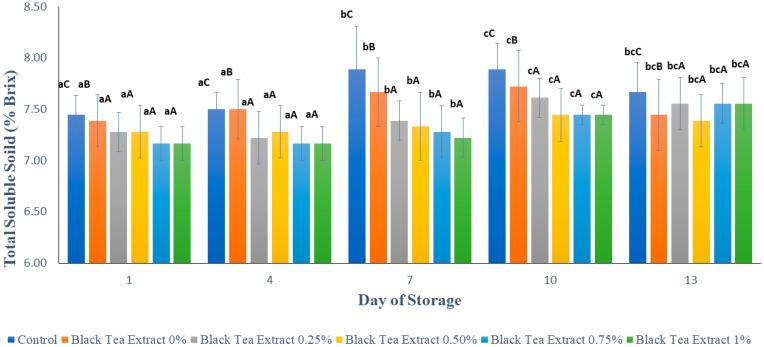
Effect of edible coating of gelatin composite enriched with black tea extract on total soluble solids (% Brix) of minimally processed watermelon during storage. The value shown is the average of the three experimental replications. ^A–C^ Values followed by the same capital letters showed no significant difference (*p* > 0.05) between treatments on the same day. ^a–c^ Values followed by the same non-capital letters showed no significant difference (*p* > 0.05) between storage times in the same treatment.

Figure 8 shows the results of the analysis of total soluble solids in minimally processed watermelon coated with an edible coating of a gelatin composite enriched with black tea extract. Total dissolved solids can be used to interpret the amount of sugar in the material and can be used as an indicator of the level of fruit maturity. The total dissolved solids values in all the treatments tended to increase significantly during the storage period (*p* < 0.05), and on the 13th day of storage, there was a slight decrease, as shown in Figure 8. However, the increase in the total dissolved solids values in the treated sample’s edible coating was not as high as in the sample without treatment (control). This could be because the coating can inhibit the rate of metabolism of the polysaccharides, allowing the sugar content in the fruit to be maintained [18].

During storage, the sugar content in fruit tends to increase due to the fruit ripening process; however, there is also a decrease in sugar content due to the use of sugar for fruit respiration and the activity of microorganisms. Chitosan has antimicrobial properties, and the combination of gelatin with chitosan can increase the antimicrobial properties of chitosan [27,28,29]. In addition, the addition of black tea extract can also increase the antimicrobial properties of the edible coating because tea has antimicrobial activity. Figure 7 shows that the addition of black tea extract to the edible coating solution affected the total soluble solids value in the minimally processed watermelon samples stored at a temperature of ±4 °C for 13 days, but there was no significant difference (*p* > 0.05) with the addition of 0.25–1% black tea extract. Often the studies involving the coating of fruit indicate that the TSS increases over time. One obvious reason for this increase is the progress in the ripening process leading to increased TSS. However, another justification for this increase is associated with the fact that water loss happens during the storage, which raises the TSS; this is a reason which has been cited in several studies [23].

### 3.6. Antioxidant Activity

**Figure 9:**
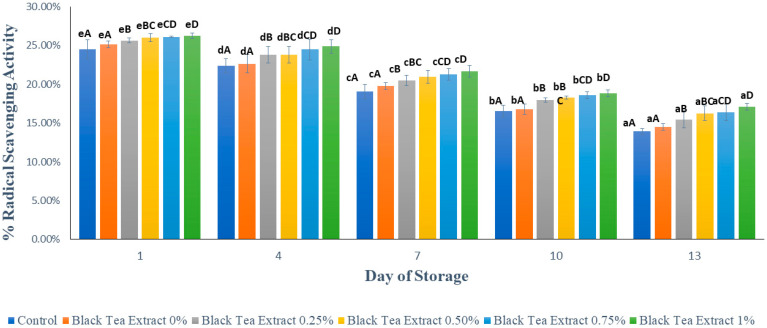
Effect of edible coating of gelatin composite enriched with black tea extract on antioxidant activity (%) of sliced watermelon during storage. The value shown is the average of the three experimental replications. ^A–D^ Values followed by the same capital letters showed no significant difference (*p* > 0.05) between treatments on the same day. ^a–e^ Values followed by the same non-capital letters showed no significant difference (*p* > 0.05) between storage times in the same treatment.

Figure 9 shows the results of the analysis of the antioxidant activity in minimally processed watermelon coated with an edible coating of a gelatin composite enriched with black tea extract. Based on Figure 9, there was a significant decrease in antioxidant activity (*p* < 0.05) in all the treatments during the storage period. The storage duration of fruits and vegetables affects their antioxidant activity: the longer the storage period, the lower the antioxidant activity due to the respiration processes [12]. The preparation and storage process can affect antioxidant activity in minimally processed watermelon samples because antioxidant compounds are sensitive and easily damaged when exposed to oxygen and light. In this study, the samples were stored for 13 days in transparent plastic cups that were translucent so that they could damage the antioxidant compounds contained in them.

In the samples without treatment (the control), the decrease in antioxidant activity was higher than that of the samples treated with the edible coating because the diffusion of O_2_ into the tissue could not be inhibited. Antioxidant compounds are sensitive to oxygen, light, and high temperatures. Gelatin as a component of edible coatings has good barrier properties against oxygen and light; so, it can reduce the decrease in antioxidant activity during storage.

The addition of black tea extract to the edible coating solution can increase the antioxidant activity of the sample, as shown in Figure 9, because tea is rich in antioxidants. One of the hurdles in the food industry is the limited shelf life of foods due to the oxidation or degradation phenomenon. The addition of tea extract provides natural antioxidant compounds, and their addition to coatings could improve their functional characteristics, making them more effective in protecting fruits [23]. Many researchers have studied how edible coatings incorporated with antioxidants affect the quality and preservation of fresh/fresh-cut fruits. The addition of tea extracts is one of the several types of antioxidants used in coatings for fresh-cut fruits. The catechin, theaflavin, and thearubigin compounds in black tea have strong antioxidant abilities so that the higher the concentration of black tea extract added, the greater the antioxidant activity [14]. Antioxidant compounds can fight free radicals and increase tissue resistance so that they can reduce damage and decay due to fungal growth. Thus, the content of antioxidant compounds can affect the shelf life of the fruit. High antioxidant activity can increase the shelf life of fruit [5].

### 3.7. Fungal Decay

**Figure 10:**
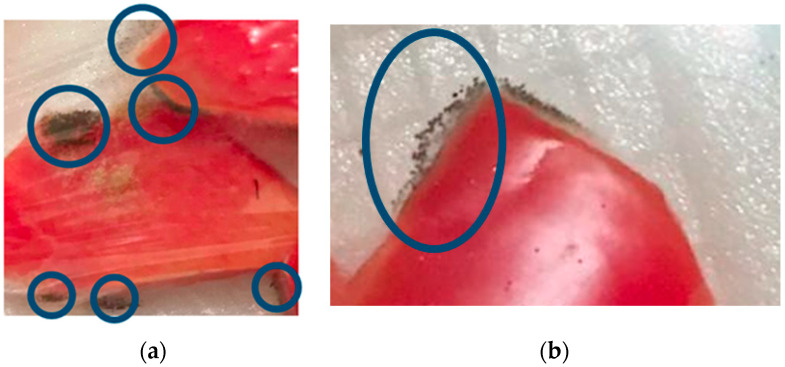
Fungal decay on the 2nd day of storage at room temperature. (±25 °C) (**a**) and the 15th day of storage at low temperature (±4 °C) (**b**).

Our investigation showed that in all the treatments no samples were found that were damaged by fungal contamination during 13 days of storage at a temperature of ±4 °C. In this study, the selection of low temperature (±4 °C) and storage time for 13 days was motivated by previous experiments, which can be seen in Figure 10. Figure 10 shows that there was damage to the sample due to fungal growth on the 2nd day of storage at room temperature (±25 °C) and on the 15th day of storage at low temperature (±4 °C). Storage at low temperatures (±4 °C) can inhibit fungal growth. Previous research showed that ciplukan fruit (Physalis peruviana), coated with an edible coating of gelatin and stored at ±5 °C, had a shelf life of up to 21 days and a lower percentage of damage due to fungal growth when compared to fruit storage at ±20 °C [30]. Storage at a temperature of ± 4 °C in minimally processed watermelon coated with an edible coating of sodium alginate can effectively reduce fungal growth until the 15th day [3].

The antimicrobial nature of the edible coating may be due either to its inherent features or to an antimicrobial agent that has been added to the coating formulation, such as chitosan [29,31]. With reference to the literature, the antimicrobial potential of essential oils is the major reason for the incorporation of essential oils into edible coatings applied to fruits. The increased consumption of fresh-cut produce in recent times has raised the interest in antimicrobial coatings. The addition of plant extracts, such as tea extract, to edible coating formulations can improve the functionality of coatings in safeguarding fruits and vegetables from microbiological spoilage and hence extend their shelf life [23].

Fresh-cut fruits such as watermelon are convenient and ready-to-eat products that deliver advantages to consumers. However, the development of novel technologies to maintain the quality and extend the shelf life of fresh-cut fruits is a major challenge for the food industry and an issue of concern for future research. The use of edible coatings helps to maintain the quality of fresh-cut watermelon and extends its shelf life. In addition, coatings can be used to incorporate active/functional ingredients such as black tea into the fresh-cut produce. They can also perform effectively as carriers of bioactive compounds.

## 4. Conclusions

Treatment with an edible coating of tuna fish skin gelatin composite and chitosan enriched with the addition of black tea extract can inhibit weight loss and texture softening, as well as provide a protective effect against the discoloration of minimally processed watermelon which is stored for 13 days at ±4 °C. An edible coating treatment of tuna skin gelatin composite and chitosan enriched with the addition of black tea extract can inhibit acidity reduction and provide a protective effect against the changes in sugar content and the antioxidant activity of minimally processed watermelon which is stored for 13 days at a temperature of ±4 °C. Treatment with an edible coating of tuna fish skin gelatin composite and chitosan enriched with the addition of black tea extract can provide a protective effect against damage due to fungal contamination in minimally processed watermelon which is stored for 13 days at a temperature of ±4 °C.

## Data Availability

Not applicable.
